# Who Benefits More in Group-Based and Individual Telehealth Parenting Programs: Moderators and Distal Child Outcomes among Military Families

**DOI:** 10.1007/s10802-026-01496-1

**Published:** 2026-08-26

**Authors:** Carlie J. Sloan, Qiyue Cai, Abigail H. Gewirtz

**Affiliations:** 1 Department of Psychology, Arizona State University, Tempe, AZ USA; 2 Reach Institute, Arizona State University, 900 S McAllister Ave, Room 205, Tempe, AZ USA; 3 Paul Baerwald School of Social Work & Social Welfare, Hebrew University of Jerusalem, Jerusalem, Israel

**Keywords:** Parenting program, Telehealth, Military, Comparative effectiveness, Moderated mediation

## Abstract

Telehealth has emerged as a feasible means for delivering evidence-based parenting interventions, but studies of which families benefit from individual telehealth versus traditional in-person groups, as well as long-term effects for children, are lacking. In this comparative effectiveness trial, 166 military families were randomized to the individual telehealth or in-person group-based ADAPT intervention. Moderated mediation models tested whether baseline parent and child factors moderated the impact of delivery format on parenting at a one-year follow-up and whether parenting changes mediated child adjustment changes at a two-year follow-up. Both mothers and fathers who perceived intitially lower child maladjustment problems showed greater gains following the in-person group condition as opposed to the telehealth condition. Fathers with higher initial mental health risk and perceived child maladjustment problems showed greater gains in the individual telehealth condition as opposed to the in-person group condition. Improvements in mothers’ parenting were associated with reductions in child internalizing and externalizing symptoms at two-year follow-up. Findings may guide tailored intervention delivery and scale-up of ADAPT and related programs.

**Supplementary Information:**

The online version contains supplementary material available at https://doi.org/10.1007/s10802-026-01496-1.

## Introduction

Evidence-based parenting interventions (or parenting programs) are effective for preventing child behavioral and emotional problems. Theoretically-grounded parenting programs have documented long-lasting positive impacts on parenting and child development (Costantini et al., 2023; Mingebach et al., 2018; Weber et al., 2019). Parenting programs based on the social interaction learning (SIL) model—particularly the Oregon Model of Parent Management Training, now known as GenerationPMTO—have been shown to reduce internalizing and externalizing problems among children presenting with or at risk for antisocial behavior problems, such as aggression, delinquency, and conduct problems (Cai et al., 2022; Forgatch & Gewirtz, 2017; Patterson, 2005).

## The ADAPT Program for Military Families

Military parents and their children stand to benefit from evidence-based parenting programs. Parents deployed in wartime conflicts may experience increased depressive symptoms and post-traumatic stress symptoms (PTSS; Creamer et al., 2011; Hoge et al., 2006). The military family stress model posits that deployment-related stressors, like length and number of deployments and parents’ mental health challenges, increase risk to children by disrupting parents’ emotion regulation capacities and undermining family processes like parenting (Gewirtz et al. 2018b). The military family stress model has been supported empirically: in families where parents had returned to the U.S. from deployment in the post-9/11 conflicts, parents’ PTSS were associated with less effective parenting, and poorer parenting was associated with poorer child adjustment (Cheng et al. 2024; Gewirtz et al. 2018b). The Adaptive Parenting Tools program (ADAPT), was developed for military families with school-aged children (Gewirtz et al., 2011). It was developed as a 14-session, in-person group-based program. ADAPT incorporates the core components of GenPMTO, as well as additional content developed specifically for military families. In a randomized controlled trial of 336 families, parents in ADAPT groups showed improvements in observed parenting one year after baseline, which mediated improvements in child adjustment two years after baseline (Gewirtz et al. 2018a).

### The Rise of Telehealth Programs

In-person, group-based programs have long been the standard in parent training interventions. Several factors, including the onset of the COVID-19 pandemic, a growing recognition that a one-size-fits-all approach to prevention is inappropriate, and analyses of the barriers that inhibit families’ access to evidence-based programming, have led program providers to increasingly look to alternative delivery formats. Digital, synchronous provider-delivered programs (i.e., telehealth programs) have emerged as a feasible and acceptable means of delivering evidence-based intervention content to parents (Howland et al., 2025; McLean et al., 2021; Pan et al., 2023). The feasibility and effectiveness of telehealth programs have been supported in family-based treatment and preventive settings, such as well-child visits, with telehealth showing non-inferioriority to face-to-face delivery in terms of effects on parent mental health, parenting stress, and child behavioral outcomes (Gewirtz et al., 2024; Howland et al., 2025; McLean et al., 2021). Evidence supporting the effectiveness of telehealth behavioral parent training programs is scarce but growing. Telehealth formats of parent-child interaction therapy, the emotion socialization program Tuning Into Kids, as well as the ADAPT program for military families have documented high attendance rates, improvements in program-targeted parenting behaviors, and there is some evidence of reductions in child behavioral problems (Burkhardt et al., 2024; Cai et al., 2024; Gewirtz et al., 2024; Peskin et al., 2024; Sanchez et al., 2024), providing promising initial evidence.

## ADAPT4U: A Comparative Effectiveness Approach

Following the effectiveness trial of the in-person, group-based ADAPT program, an individual telehealth format of the program was developed wherein families met one-on-one with a trained facilitator. The individual telehealth and group-based in person formats, in addition to a fully online self-guided format, were tested in a comparative effectiveness trial, *ADAPT4U* (Gewirtz et al., 2024). Findings corroborated existing evidence about the effectiveness of telehealth delivery. For one, using intent-to-treat analyses, the indiv

idual telehealth and in-person group conditions were both found to be superior, on average, to the online condition in terms of improvements in parenting one year after baseline (Gewirtz et al., 2024), and parents rated both conditions as highly acceptable overall (Buchanan et al., 2025). These findings underscore the importance of features like provider interaction in parenting programs for most families. Of note, Chan and colleagues (2026) found that for low risk families, the online format was equally effective or even superior to the provider-facilitated formats. Key differences between the individual telehealth and in-person group formats may help explain who benefits more from each type of program. Despite both formats having the same content and number of sessions, parents in the telehealth condition showed higher overall enrollment (75%, compared to 54% for in-person groups) and sustained attendance rates (Buchanan et al., 2025; Cai et al., 2024). In contrast, those in the in-person group condition were more satisfied with specific weekly sessions (Buchanan et al., 2025). These findings may reflect the key differences between the group and telehealth formats, like opportunities for peer support in the group condition versus one-on-one provider attention in the telehealth condition. Thus, the different program modalities likely meet the needs and wants of different families.

Understanding who benefits most from different intervention delivery modalities is a key next step toward personalizing intervention approaches for diverse families. The military family stress model suggests that parents with initially higher risk (e.g., greater initial child behavior problems, higher parent PTSS, poorer parent mental health) are at greater risk for parenting deficits (Gewirtz et al., 2018b). As documented in many in-person parenting programs, parents at greater initial risk tend to show greater improvements in parenting following interventions, likely because they had more to gain (see Gardner, 2023). This compensatory effect has not been extensively studied in telehealth interventions for parents, but is an important step in determining whether the mechanisms of intervention effects are similar across face-to-face and telehealth modalities. It may be that the one-on-one attention provided in the telehealth condition serves to benefit families at higher initial risk, whereas in-person formats result in less personal attention.

Furthermore, it is important to understand the extent to which program-driven effects on parenting serve to prevent downstream child maladjustment problems. Parenting programs are predicated on the idea that improvements in parenting mediate distal improvements to child maladjustment problems. This model of change has been supported in in-person group-based parenting programs, especially preventive interventions (see Forehand et al., 2014 for review), but research on telehealth programs lags behind. The extant data has suggested similar effectiveness of telehealth and in-person programs, as well as advantages of telehealth in terms of cost, efficiency, and reach (Cai et al., 2024; Stover et al., 2024). However, much of the research has been on short-term impacts (e.g., within 6 months of intervention delivery). To fully understand the potential benefits of telehealth parenting programs, we must determine whether they provide at least non-inferior long-term downstream benefits to children as in-person programs.

## The Current Study

This study utilized a moderated mediation approach to explore two research aims: to determine whether baseline parent depressive symptoms, PTSS, and child adjustment problems moderated the effects of individual telehealth or in-person group-based conditions of ADAPT on parenting outcomes (Aim 1), and whether changes in parenting mediated changes in child adjustment two years later for telehealth and group-based ADAPT conditions (Aim 2). Whereas a prior investigation established the non-inferiority of the individual telehealth and in-person group conditions of ADAPT for parenting outcomes at one year post-baseline (Gewirtz et al., 2024), we aimed to understand whether some families benefit more from each condition than others, as well as downstream program effects on child adjustment. Given the dearth of randomized comparisons of telehealth and in-person parenting interventions, especially from trials other than the current one, we refrained from making specific hypotheses and considered these analyses to be exploratory.

## Method

### Participants

A total of 244 families (223 mothers, 193 fathers, one target child per family) participated in the ADAPT4U comparative effectiveness trial, in which National Guard and Reserve families were randomly assigned to one of three ADAPT program conditions: in-person groups, individual telehealth, or self-directed online. Families were recruited from two Midwestern states. Families were eligible to participate if they: (1) had a parent who had returned from deployment to Operation Enduring Freedom, Operation Iraqi Freedom, or from stateside mobilization in support of the Global War on Terror, and (2) had at least one child between the ages of 5 and 12 years old who lived in their home. For families with multiple children in the age range, only one target child was randomly selected to be involved in the study. More details about the study design and demographic information can be found in Gewirtz et al. (2024).

The current analytic sample consisted of 166 families (148 mothers and 128 fathers) who were assigned to either the in-person group (*n* = 95) or individual telehealth (*n* = 71) condition. Mothers were on average 36.5 years old (*SD* = 5.7, range = 26–54) and fathers were 38.5 (*SD* = 5.8, range = 25–54). Target children (52.4% girls) were on average 7.8 years old (*SD* = 2.3). Most families (80.7%) comprised dual White/European American parents; 19.3% of families had at least one parent of color. Most parents reported being married or cohabitating (86.4% of mothers, 87.4% of fathers). Of the 166 families in the current sample, 110 were in couples (mothers and fathers participating together), whereas the remaining 56 either only had one parent participating (partner declined to participate) or were single mothers or fathers. Annual family income ranged from *less than $25*,*000* to *greater than 151*,*000*, with a median income of *$51*,*000–80*,*000*. In terms of deployment status, 94.5% of fathers and 26.5% of mothers had been deployed. The modal number of deployments was 2 times for fathers (32.8%; range = 1 to 10), and one time for mothers (66.7%; range = 1 to 6); 15.1% of fathers and 5.1% of mothers had been deployed more than 3 times.

### Procedures

Participants were recruited in partnership with National Guard and Reserve Units, Veterans Affairs Medical Centers, and other military family organizations in two Midwestern states. Recruitment methods included flyer distribution, project staff outreach through presentations in schools and community spaces, and word of mouth. One or both parents from a family were invited to participate; participants could be military parents and/or their civilian partner. After completing an eligibility screener, parents provided informed consent via a secure online form and were invited to complete a baseline protocol that included an online survey and a home visit (Time 1/T1). The home visits included additional self-report questionnaires as well as video-recorded parent-child interactions. Families were then randomized to one of the three ADAPT program conditions described above. In the current study, we utilized the sample of parents randomized to the telehealth or in-person group conditions.

In-person parenting groups consisted of 14 once-weekly sessions led by 2–3 ADAPT facilitators; each included 5–10 families. Sessions were two hours long. They took place in community spaces such as local schools or libraries, selected based on geographic proximity to the participants. The telehealth condition consisted of 14 once-weekly individual family sessions (i.e. 1–2 parents) led by one ADAPT facilitator. Sessions lasted one hour each and were administered through the videoconferencing platform WebEx. Both the individual telehealth and in-person group conditions covered the same content, i.e., key parenting skills: positive involvement, skill encouragement, problem solving, effective discipline, monitoring, and emotion socialization of children (e.g., emotion regulation and emotion coaching). Session activities include psychoeducation about core parenting skills, mindfulness exercises, role play, discussion, and home practice assignments. Families in both the telehealth and in-person group conditions had access to the self-guided online modules, which included videos and handouts, to use for additional practice or to review content. In both conditions, ADAPT facilitators called families weekly to address questions and troubleshoot home practice assignments. Participants completed an additional online survey and home visit protocol one year after baseline (T3) and two years after baseline (T4). Data collected 6-months after baseline (T2) did not include a home visit and therefore were not used in the current study. The procedures used in this study were approved by the Institutional Review Board at the University of Minnesota (study no. 1407S52001). The study was registered at https://www.clinicaltrials.gov (NCT03522610; Unique Protocol ID: 1005S82692).

### Measures

Descriptive statistics, Cronbach’s *α* reliability coefficients, and correlations for study variables are shown in Table 1.

**Table 1:** Correlations and descriptive statistics

	1	2	3	4	5	6	7	8	9	10	11	12	13	14	15
1. Condition	--	0.16	− 0.02	− 0.08	− 0.05	− 0.01	0.19	− 0.02	− 0.02	0.01	0.08	0.03	− 0.03	− 0.14	− 0.01
2. T1 Obs. Parenting	− 0.06	--	**− 0.23**	0.14	− 0.09	− 0.13	**0.43**	− 0.06	− 0.06	− 0.13	− 0.04	− 0.10	0.12	0.07	0.06
3. T1 PTSS	0.10	− 0.02	--	0.14	**0.32**	**0.27**	− 0.18	**0.39**	**0.24**	0.13	0.09	0.13	**− 0.36**	**− 0.22**	0.08
4. T1 Depression	− 0.04	0.03	0.11	--	0.07	0.13	0.08	0.02	0.09	0.05	− 0.01	− 0.01	0.04	0.07	**0.81**
5. T1 Child Internalizing	0.02	0.12	**0.25**	0.06	--	**0.49**	− 0.08	**0.64**	**0.34**	0.03	0.12	− 0.04	**− 0.19**	**− 0.16**	**0.22**
6. T1 Child Externalizing	0.04	**− 0.14**	**0.26**	0.06	**0.41**	--	0.02	**0.39**	**0.75**	− 0.06	− 0.07	− 0.09	− 0.13	0.04	**0.21**
7. T3 Obs. Parenting	0.07	**0.31**	− 0.01	− 0.16	0.17	− 0.09	--	− 0.16	− 0.09	− 0.09	− 0.07	− 0.00	0.07	0.11	0.02
8. T4 Child Internalizing	0.07	− 0.04	0.14	0.09	**0.52**	0.15	− 0.04	--	**0.59**	0.13	**0.19**	0.11	− 0.15	− 0.03	0.17
9. T4 Child Externalizing	0.08	**− 0.19**	0.11	− 0.03	**0.24**	**0.63**	**− 0.19**	**0.46**	--	− 0.08	− 0.11	0.00	− 0.03	0.01	**0.18**
10. T1 Child Age	0.01	**− 0.17**	0.09	− 0.11	− 0.03	− 0.06	**− 0.20**	**0.20**	0.07	--	0.06	**0.13**	− 0.08	**− 0.16**	0.08
11. Child Sex (1 = female)	0.08	− 0.13	0.13	− 0.05	0.13	− 0.08	− 0.15	**0.19**	− 0.01	0.06	--	0.07	− 0.07	− 0.01	− 0.01
12. Site (coded as 1 or 2)	0.03	**− 0.18**	− 0.03	**− 0.15**	− 0.13	− 0.02	− 0.03	− 0.05	0.03	**0.13**	0.07	--	− 0.16	− 0.05	− 0.01
13. Income	− 0.03	**0.17**	**− 0.18**	0.02	− 0.03	**− 0.16**	− 0.04	− 0.09	**− 0.18**	− 0.05	− 0.04	**− 0.21**	--	**0.27**	− 0.06
14. Married (1 = yes)	− 0.02	0.05	**− 0.24**	0.05	0.01	0.03	0.02	0.06	0.06	− 0.02	0.01	− 0.12	**0.35**	--	− 0.04
15. No. of Deployments	0.01	0.00	**0.20**	**0.21**	0.09	0.05	− 0.00	0.08	− 0.01	− 0.12	0.01	− 0.05	− 0.04	**− 0.36**	--
Mean (Mothers)		2.64	28.57	12.82	52.49	52.57	2.64	50.36	51.25	7.7	0.52	1.28	$51-80k	0.86	3.12
Mean (Fathers)		2.47	35.84	14.53	51.30	52.89	2.45	49.46	52.40					0.34	6.24
SD (Mothers)		0.36	10.77	11.66	12.05	10.83	0.33	11.91	9.97	2.3	0.50	0.45		0.87	3.52
SD (Fathers)		0.39	16.73	12.53	11.14	11.13	0.46	11.11	10.80					0.33	4.37
Cronbach’s$$\:\alpha\:$$(Mothers)		0.56-0.87	0.93	0.94	0.73	0.86	0.54-0.86	0.75	0.86						
Cronbach’s$$\:\alpha\:$$(Fathers)		0.59-0.89	0.97	0.94	0.70	0.84	0.54-0.87	0.71	0.85						

Correlations for fathers are shown above the diagonal and for mothers below the diagonal; bolded correlations are statistically significant at the *p* < .05 level; Obs. = Observed, PTSS = Post-Traumatic Stress Symptoms, No. = Number

#### Observed Parenting

Observed parenting was measured at Time 1 (baseline) and Time 3 (one-year follow-up) using scores obtained through observed parent-child interactions during structured family interaction tasks (FITs). FITs consisted of a set of 5-minute tasks for parents and children, including discussing a source of everyday conflict, discussing deployment-related concerns, planning a fun family activity, a teaching activity in which parents provided assistance to their child in a game, and a monitoring activity in which parents solicited information from their child about a time when they (parents) were not present. Trained observers reviewed video-recorded FITs and scored them using an established coding system (Forgatch et al., 1992). FIT codes have been extensively validated in both military and at-risk families (see Gewirtz et al., 2024 for more details). Five subscales based on SIL constructs comprised the observed parenting scale: (1) problem-solving outcome, (2) harsh discipline, (3) positive involvement, (4) skill encouragement, and (5) monitoring.

Scores for problem-solving (9 items) were based on coders’ ratings of the extent of resolution, quality of the solution, apparent satisfaction with the solution, and perceived likelihood that the family would enact the solution (*α*_mothers, T1_=0.87, *α*_fathers, T1_=0.86, *α*_mothers, T3_ =0.83, *α*_fathers, T3_=0.87). Harsh discipline (8 items) included ratings of parents’ overly strict, erratic, authoritarian, or inconsistent parenting (*α*_mothers, T1_=0.70, *α*_fathers, T1_=0.77, *α*_mothers, T3_ =0.54 *α*_fathers, T3_=0.54). Positive involvement (10 items) included ratings of parents’ warmth, empathy, encouragement and affection (*α*_mothers, T1_=0.87, *α*_fathers, T1_=0.89, *α*_mothers, T3_ =0.86 *α*_fathers, T3_=0.86). Skill encouragement (8 items) assessed parents’ ability to use encouragement and scaffolding to promote children’s skill development (*α*_mothers, T1_=0.80, *α*_fathers, T1_=0.81, *α*_mothers, T3_ =0.72 *α*_fathers, T3_=0.78). Monitoring (4 items) assessed parents’ supervision and knowledge of children’s daily activities (*α*_mothers, T1_=0.56, *α*_fathers, T1_=0.59, *α*_mothers, T3_ =0.66 *α*_fathers, T3_=0.66). The poorer reliabilities of the harsh discipline and monitoring subscales are likely due to lower occurrence of these behaviors. The five indicators of observed parenting were averaged, with the harsh discipline score reverse-coded, for an overall composite parenting score at each timepoint. All subscales were included in the total composite score in order to have a holistic representation of parenting, to be consistent with the coding scheme (Forgatch et al., 1992) and with prior studies. Each subscale was positively correlated with other subscales and with the total composite score for both mothers and fathers at each timepoint (*r*s 0.27 to 0.80).

#### Child Adjustment

Parents reported on adjustment using the Internalizing Problems and Externalizing Problems subscales of the Behavioral Assessment Scale for Children (BASC-2; Raynolds & Kamphaus, 2004) at Time 1 (baseline) and Time 4 (two-year follow-up). Parents rated the frequency of child problem behaviors on a scale from 0 (never) to 3 (almost always). Internalizing is a composite of three subscales: depression, anxiety, and somatization. Externalizing is a composite of three subscales: hyperactivity, aggression, and conduct problems. Items vary across three age-specific versions of the measure, for children aged 5–7, 8–11, and 12–18. Age-normed *T* scores were calculated according to the BASC 2 scoring manual, to standardize scores across the different child ages represented in the sample.

#### Parental Depression

Parents reported their depressive symptoms in the past week using the Center for Epidemiologic Studies Depression Scale (CES-D; Radloff, 1977). The scale consists of 20 items (e.g., “I felt sad,” “I felt that everything I did was an effort”) which parents rated on a scale from 0 [Rarely or none of the time (less than 1 day)] to 3 [Most or all of the time (5–7 days)]. Items were summed such that higher scores reflected greater depressive symptoms.

#### Parental Post Traumatic Stress Symptoms

Parents completed the 17 items of the Post-Trumatic Stress Disorder (PTSD) Checklist (PCL; Weathers et al., 1993), which assesses post-traumatic stress symptoms (e.g., “Repeated, disturbing memories, thoughts or images of a stressful [experience from the past / military experience]) in the past month on a scale from 1 (Not at all) to 5 (Extremely). There are two versions of the PCL, one for previously deployed personnel (PCL-M) and one for civilians (PCL-C), which parents completed based on their previous deployment status Items were summed to compute a total score, with higher scores indicating more severe PTSD symptoms.

#### Covariates

Based on theory and prior empirical findings, we included the following covariates: child age at T1 (in years), child sex (male or female), state/site (one of two midwestern states), household income, marital status (0 = single/divorced/widowed, 1 = married/cohabiting), and parents’ reported number of deployments.

### Analytic Strategy

**Fig. 1:**
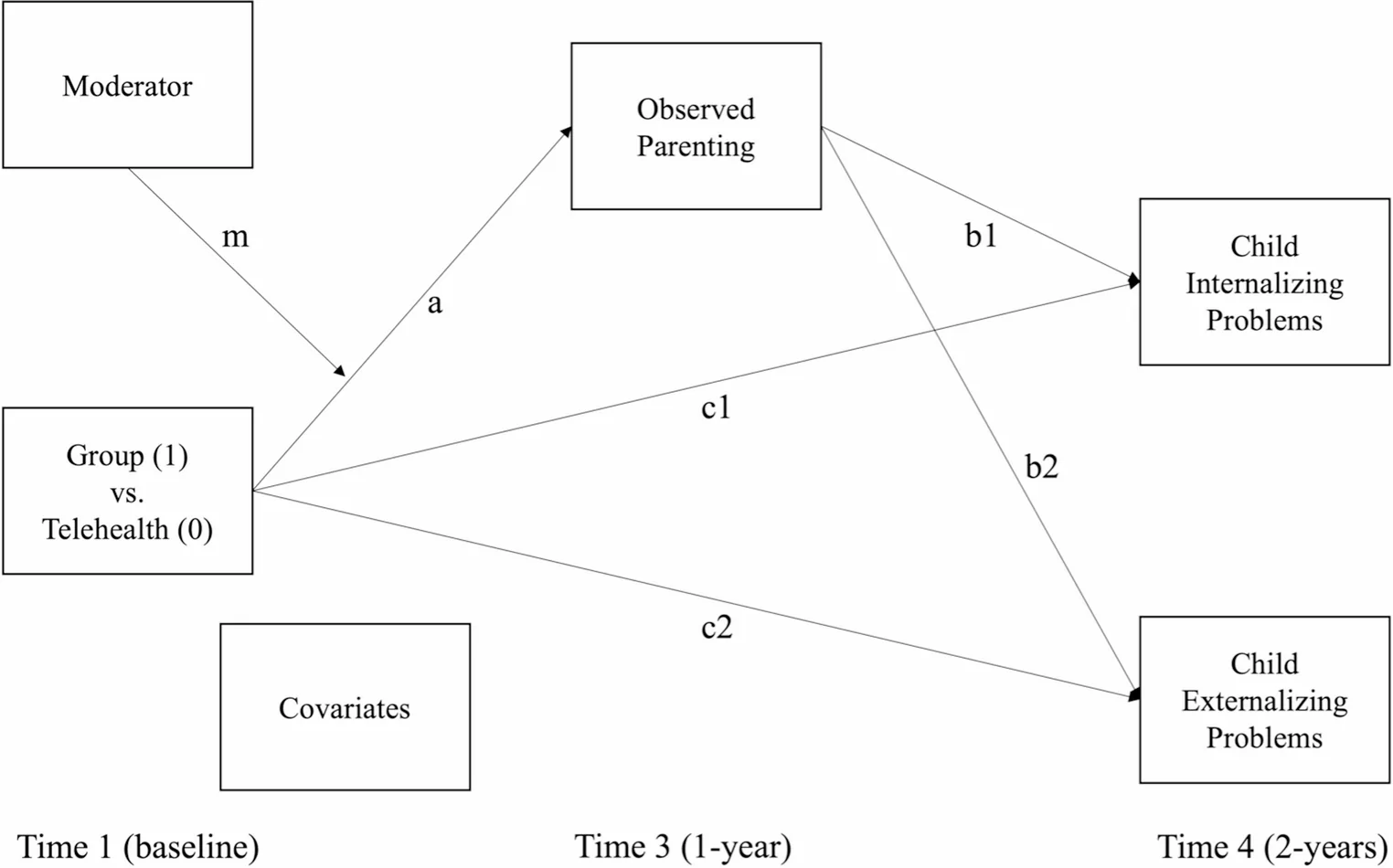
Prototypical moderated mediation model. Separate models were run for mothers and fathers. Covariates: child age, child gender, intervention site, income, marital status, number of combat experiences

Moderated mediation models were conducted in which intervention condition (0 = telehealth, 1 = group) was the independent variable, observed parenting at T3 was the mediator, and child internalizing and externalizing problems at T4 were outcomes, controlling for baseline parenting and child maladjustment problems. Separate moderated mediation models were conducted for four different moderators: baseline parental depression, baseline parental PTSS, baseline child internalizing and externalizing problems. Figure 1 depicts a prototypical model. We conducted models for mothers and fathers separately as some families only had one parent participating. Paternal and maternal parenting were only moderately correlated (T1 *r*=.45, T3 *r*=.38), and there were significant differences between paternal and maternal parenting at T1 and T3 (paired t-tests *p* < .001). Covariates included baseline levels of moderators, child age at T1, child sex, state/site, household income, marital status, and reported number of combat experiences. Correlations and descriptive statistics for all study variables are shown in Table 1. All analyses were conducted using Mplus 8.8 (Muthen & Muthen, 1998–2017). Little’s (1988) missing completely at random (MCAR) assumption was not met: χ2(1305) = 1701.06, *p* < .001. Full Information Maximum Likelihood (FIML), which addresses data that are missing at random (Enders, 2022) was used in Mplus 8.8. The maximum likelihood robust (MLR) estimator was used in model estimation.

## Results

### Aim 1: Group and Telehealth Program Effects on Parenting

**Fig. 2:**
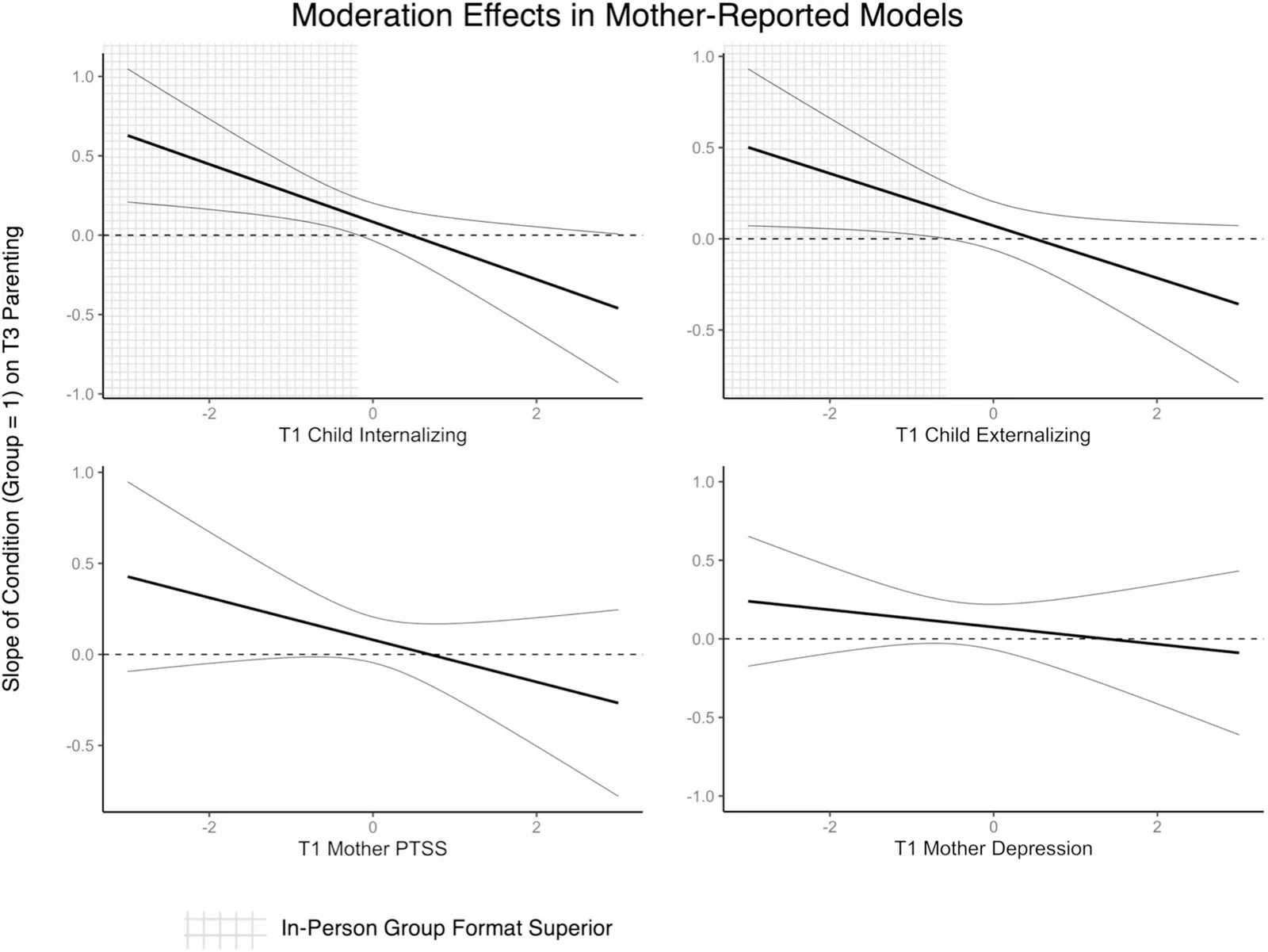
Johnson-Neyman plots of the association between intervention condition (Group = 1, Telehealth = 0) and mothers’ Time 3 parenting, at different levels of moderator variables. Models controlled for autoregressive paths as well as child age, child gender, intervention site, income, marital status, and number of deployments. PTSS = Post-Traumatic Stress Symptoms

Across mother and father models, the effect of in-person group versus individual telehealth condition on T3 parenting was not statistically significant (*p*s = 0.15 to 0.36), suggesting non-inferior main effects of the two conditions on T3 parenting. The main outcomes evaluation of this comparative effectiveness trial (Gewirtz et al., 2024) had established non-inferiority of the individual telehealth and in-person group conditions in terms of effects on T3 parenting. The current study focused on moderators of effectiveness. In mother-report models (Table 2), the association between intervention condition and T3 parenting was moderated by child baseline internalizing (*p*=.006) and externalizing (*p*=.037). When baseline child internalizing and externalizing were low, the in-person group condition was associated with greater improvements in maternal parenting than the individual telehealth condition (Internalizing: Slope_−1SD_=0.27, SE=0.08, *p*=.002; Externalizing: Slope_−1SD_=0.22, SE=0.10, *p*=.026). There were no differences between the program formats when baseline child internalizing and externalizing were high. Mothers’ depression (*p*=.449) and PTSS (*p*=.163) did not moderate effects of intervention condition on T3 parenting. Moderation effects are shown in Fig. 2.

**Table 2:** Results from moderated mediation models among mothers

Model Fit	Moderator							
	Mother Depression		Mother PTSS		Child Internalizing		Child Externalizing	
Chi Square	32.56 (*p* = .051)		32.13 (*p* = .057)		14.53 (*p* = .486)		17.58 (*p* = .285)	
CFI	0.915		0.922		1.000		0.982	
TLI	0.842		0.854		1.000		0.956	
RMSEA [95% CI]	0.058 [0.00, 0.09]		0.057 [0.00, 0.09]		0.00 [0.00, 0.07]		0.032 [0.00, 0.08]	
Path Coefficients	$\:\beta\:$(S.E.)	*p*	$\:\beta\:$(S.E.)	*p*	$\:\beta\:$(S.E.)	*p*	$\:\beta\:$(S.E.)	*p*
(a) Condition (Group vs. TH) ◊ T3 Parenting	0.11 (0.11)	0.335	0.11 (0.09)	0.228	0.12 (0.09)	0.176	0.10 (0.10)	0.304
(m) Moderator on path a	− 0.13 (0.17)	0.449	− 0.24 (0.17)	0.163	**− 0.40 (0.14)**	**0.006**	**− 0.31 (0.15)**	**0.037**
(b1) T3 Parenting ◊ T4 Child Internalizing	− 0.25 (0.21)	0.227	− 0.25 (0.14)	0.078	**− 0.29 (0.15)**	**0.050**	**− 0.35 (0.17)**	**0.043**
(b2) T3 Parenting ◊ T4 Child Externalizing	− 0.25 (0.15)	0.088	**− 0.30 (0.14)**	**0.033**	**− 0.30 (0.14)**	**0.025**	**− 0.32 (0.15)**	**0.033**
(c’1) Condition ◊ T4 Child Internalizing	0.02 (0.08)	0.832	0.02 (0.08)	0.820	0.02 (0.08)	0.787	0.03 (0.08)	0.658
(c’2) Condition ◊ T4 Child Externalizing	0.07 (0.07)	0.350	0.04 (0.07)	0.540	0.08 (0.08)	0.308	0.08 (0.07)	0.285
Autoregressive Paths								
T1 Parenting ◊ T3 Parenting	**0.30 (0.15)**	**0.047**	**0.33 (0.09)**	**< 0.001**	**0.24 (0.10)**	**0.013**	**0.31 (0.10)**	**0.003**
T1 Child Internalizing ◊ T4 Internalizing	**0.47 (0.09)**	**< 0.001**	**0.50 (0.08)**	**< 0.001**	**0.48 (0.08)**	**< 0.001**	**0.47 (0.08)**	**< 0.001**
T1 Child Externalizing ◊ T4 Externalizing	**0.55 (0.14)**	**< 0.001**	**0.55 (0.09)**	**< 0.001**	**0.55 (0.10)**	**< 0.001**	**0.58 (0.10)**	**< 0.001**

Standardized coefficients are presented; TH = Telehealth condition; Models controlled for prior levels of all dependent variables; Covariates: child age, child gender, intervention site, income, marital status, number of deployments; CFI=Comparative Fit Index, TLI=Tucker-Lewis Index, RMSEA= Root Mean Square Error of Approximation; Chi Square was tested on 21 degrees of freedom in Models 1 and 2, and 15 degrees of freedom in Models 3 and 4

**Fig. 3:**
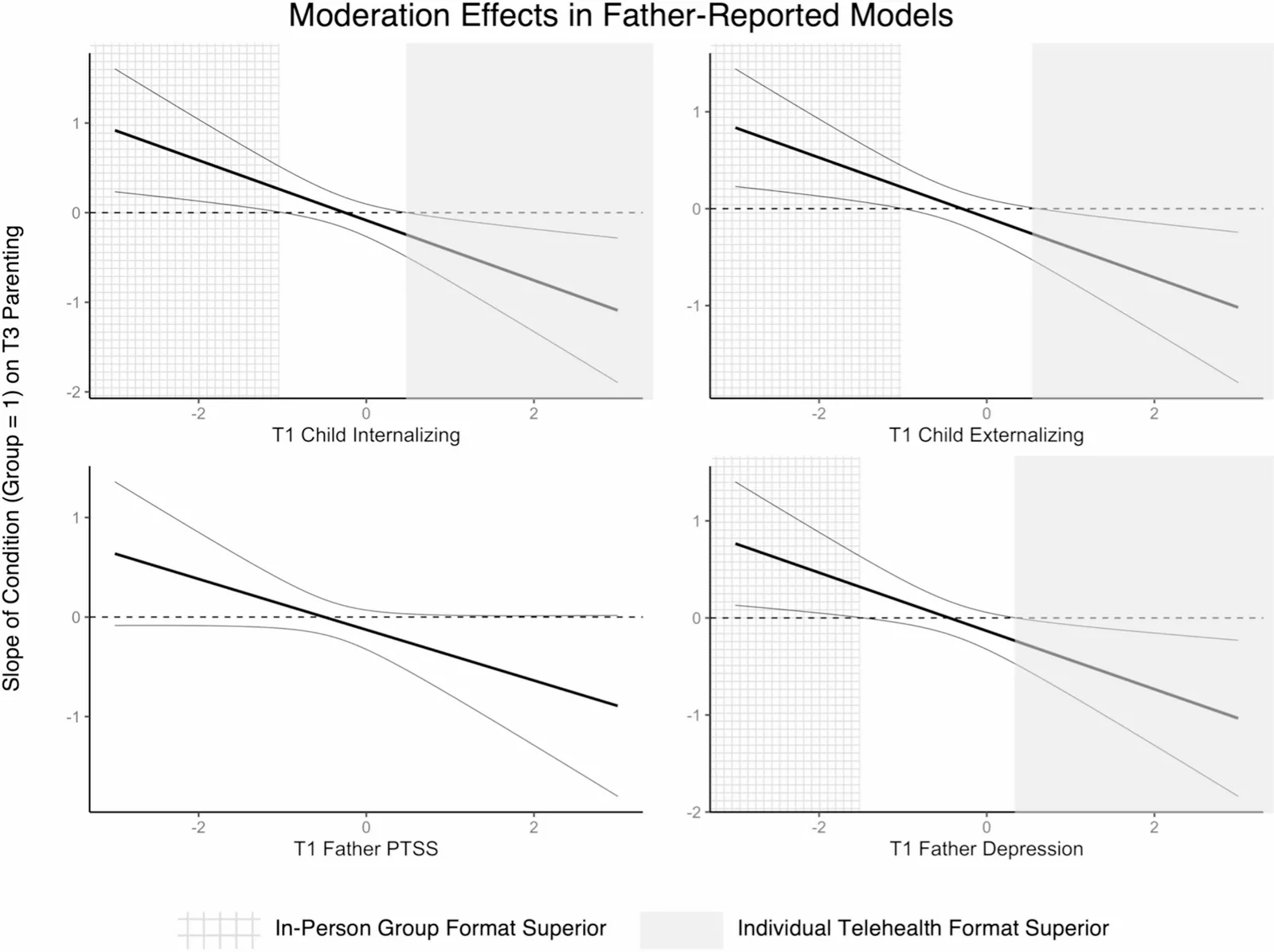
Johnson-Neyman plots of the association between intervention condition (Group = 1, Telehealth = 0) and fathers’ Time 3 parenting, at different levels of moderator variables. Models controlled for autoregressive paths as well as child age, child gender, intervention site, income, marital status, and number of deployments. PTSS = Post-Traumatic Stress Symptoms

In father-reported models (Table 3), the association between intervention condition and T3 parenting was moderated by child baseline internalizing (*p*=.001) and externalizing (*p*=.001), and father’s baseline depressive smptoms (*p*=.002) and PTSS (*p*=.025). Parallelling mother-reported models, when baseline child internalizing and externalizing were low, the in-person group condition was associated with greater improvements in paternal parenting than the individual telehealth condition (Internalizing: Slope_−1SD_=0.25, SE=0.13, *p*=.049; Externalizing: Slope_−1SD_=0.22, SE=0.11, *p*=.050). Father models also showed crossover effects: When baseline child internalizing and externalizing were high, the individual telehealth condition was associated with greater improvements in parenting than the in-person group condition (Internalizing: Slope_+1SD_=-0.42, SE=0.18, *p*=.018; Externalizing: Slope_+1SD_=-0.40, SE=0.18, *p*=.026). The same pattern of crossover effects was observed for father-reported depressive symptoms. For fathers lower in initial depressive symptoms, the in-person group condition was associated with greater improvements in parenting than the individual telehealth condition, although only for fathers whose depression was lower than 1 standard deviation below average (Slope_−1SD_=0.17, SE=0.11, *p*=.146). In contrast, for fathers higher in initial depressive symptoms, the individual telehealth condition was associated with greater improvements in parenting than the in-person group condition (Slope_+1SD_=-0.43, SE=0.19, *p*=.019). A significant moderation effect was also found for fathers’ PTSS (*p*=.025); simple slopes analysis revealed that the moderation effect was not significant within the range of observed data. We found a marginal effect in which fathers with higher initial PTSS saw greater improvements in the individual telehealth condition than the in-person group-based condition (Slope_+1SD_=-0.38, SE=0.20, *p*=.061). Moderation effects are shown in Fig. 3.

**Table 3:** Results from moderated mediation models among fathers

Model Fit	Moderator							
	Father Depression		Father PTSS		Child Internalizing		Child Externalizing	
Chi Square	28.77 (*p* = .119)		25.76 (*p* = .216)		41.84 (*p* = .000)		19.24 (*p* = .203)	
CFI	0.954		0.970		0.832		0.974	
TLI	0.914		0.945		0.612		0.939	
RMSEA	0.047 [0.00, 0.09]		0.037 [0.00, 0.08]		0.104 [0.07, 0.14]		0.041 [0.00, 0.09]	
Path Coefficients	*β*(S.E.)	*p*	*β*(S.E.)	*p*	*β*(S.E.)	*p*	*β*(S.E.)	*p*
(a) Condition (Group vs. TH) ◊ T3 Parenting	− 0.16 (0.11)	0.152	− 0.15 (0.11)	0.185	− 0.10 (0.11)	0.358	− 0.11 (0.11)	0.341
(m) Moderator on path a	**− 0.56 (0.19)**	**0.002**	**− 0.48 (0.21)**	**0.025**	**− 0.58 (0.18)**	**0.001**	**− 0.52 (0.16)**	**0.001**
(b1) T3 Parenting ◊ T4 Child Internalizing	0.01 (0.14)	0.920	− 0.03 (0.14)	0.849	0.01 (0.13)	0.943	− 0.02 (0.13)	0.851
(b2) T3 Parenting ◊ T4 Child Externalizing	− 0.10 (0.13)	0.940	− 0.03 (0.15)	0.849	− 0.04 (0.14)	0.760	− 0.01 (0.14)	0.472
(c’1) Condition ◊ T4 Child Internalizing	0.01 (0.08)	0.914	0.01 (0.08)	0.931	0.04 (0.08)	0.603	0.04 (0.08)	0.612
(c’2) Condition ◊ T4 Child Externalizing	− 0.01 (0.08)	0.891	− 0.01 (0.08)	0.916	− 0.01 (0.08)	0.863	− 0.02 (0.08)	0.812
Autoregressive Paths								
T1 Parenting ◊ T3 Parenting	**0.37 (0.13)**	**0.004**	**0.37 (0.13)**	**0.005**	**0.33 (0.12)**	**0.006**	**0.42 (0.14)**	**0.002**
T1 Child Internalizing ◊ T4 Internalizing	**0.62 (0.08)**	**< 0.001**	**0.62 (0.08)**	**< 0.001**	**0.63 (0.07)**	**< 0.001**	**0.63 (0.07)**	**< 0.001**
T1 Child Externalizing ◊ T4 Externalizing	**0.72 (0.06)**	**< 0.001**	**0.72 (0.06)**	**< 0.001**	**0.73 (0.06)**	**< 0.001**	**0.73 (0.06)**	**< 0.001**

Standardized coefficients are presented; TH = Telehealth condition; Models controlled for prior levels of all dependent variables; Covariates: child age, child gender, intervention site, income, marital status, number of deployments; CFI=Comparative Fit Index, TLI=Tucker-Lewis Index, RMSEA= Root Mean Square Error of Approximation; Chi Square was tested on 21 degrees of freedom in Models 1 and 2, and 15 degrees of freedom in Models 3 and 4

### Aim 2: Group and Telehealth Effects on Distal Child Adjustment

Models also evaluated whether improvements in parenting following ADAPT participation were associated with improvements in child adjustment outcomes two years after baseline. We did not find evidence of mediation in father-reported models. In mother-reported models, improvements in parenting at one year were associated with small-to-medium decreases in child internalizing (*β* range from − 0.35 to -0.25; *p*s 0.04 to 0.23) and externalizing (*β* from − 0.25.

to -0.32; *p*s 0.03 to 0.09) at two years post-baseline.

Of the covariates, child age was negatively associated with T3 observed parenting across most models (*β* from − 0.21 to − 0.32, *p*s 0.04 to < 0.001). Fathers’ higher number of combat experiences were associated with greater child internalizing at T4 in models with child internalizing and externalizing as moderators (*β*=0.21 in both models, *p*=.05 and 0.04), and mothers’ higher number of combat experiences were associated with lower child externalizing in the model with mother PTSS as the moderator (*β*=-0.20, *p*=.04). Child gender, intervention site, parent income, and marital status were not related to dependent variables in any models.

### Post-Hoc Analyses: Examining Effects by Parental Military Status

As a post-hoc analysis, we ran models separately for civilian versus military parents. The purpose of running additional models was because military status and parent gender are somewhat conflated, with fathers most commonly being the military deployed parent. Examining results based on military status may inform certain clinical approaches (e.g., among veterans) better than distinguishing by parent gender.

Results, which are presented in the supplemental materials (Tables S1 and S2), were largely consistent with mother- and father-report models. Among civilian parents (*N* = 115), the association between program type and T3 parenting was moderated by perceived child externalizing (*p*=.019), marginally moderated by perceived child internalizing (*p*=.066), and not moderated by parent depressive symptoms or PTSS. Moderation effects were such that improvements in T3 parenting were greater in the in-person group-based condition for those with lower initial child maladjustment problems. As in mother-report models, improvements in T3 parenting were associated with reduced child internalizing problems (although the externalizing pathway was not supported).

Among previously deployed parents (*N* = 159), two-way moderation effects emerged for baseline child internalizing (*p*=.002), externalizing (*p*=.002), and parental depression (*p*=.005), wherein when child maladjustment problems and parental depression were low, parents experienced greater improvements in parenting in the in-person group-based condition; when child maladjustment problems and parental depression were high, parents experienced greater improvements in parenting in the individual telehealth condition. Similar to father-report models, there was an overall moderation effect for parents’ PTSS (*p*=.033), wherein parents higher in baseline PTSS experienced greater improvements in parenting in the individual telehealth condition, but only at very high levels (e.g., greater than 1SD above the mean) of PTSS. Also as in father-reported models, T3 parenting was not associated with T4 child adjustment among previously deployed parents.

## Discussion

The COVID-19 pandemic prompted the rapid proliferation of alternative intervention modalities such as telehealth and self-guided programs, which are likely to persist (Thomas et al., 2022). Whereas most telehealth research to date has focused on feasibility, understanding of long-term effects and the suitability of different program formats for families with different initial risk profiles is nascent. In a randomized comparative effectiveness trial of an evidence-based parenting program for military families, ADAPT, we found that families with initially higher child maladjustment and parent mental health risk generally showed greater improvements in parenting when randomized to the individual telehealth format, whereas families with lower initial risk showed greater gains in the in-person group-based format. Furthermore, we found evidence that improvements in maternal parenting following ADAPT were associated with reductions in child internalizing and externalizing problems, supporting the program’s overall theory of change. The findings contribute to a growing literature base about the appropriateness of different intervention formats for specific families. Research disentangling the appropriateness of telehealth versus in-person as well as group-based versus individual formats for specific families is critically needed.

### Effects of Individual Telehealth Versus In-Person Group Formats on Parenting

Although parenting in both the in-person group-based and individual telehealth versions of ADAPT improved at one year post-baseline (replicating previous findings; Gewirtz et al., 2024), accounting for parents’ perceptions of initial child maladjustment and their own mental health difficulties revealed clear differences in the impacts of the two program formats. Turning first to child adjustment risk, both mothers and fathers who reported lower initial child adjustment problems showed greater gains in parenting following the in-person group-based, rather than individual telehealth, format of ADAPT.

In father-reported models, we also found evidence of crossover effects: when child internalizing and externalizing were initially high, fathers’ parenting improved more in the individual telehealth rather than the in-person group condition. The telehealth condition involved one-on-one family attention, which may be important for families who present with more child emotional and behavioral challenges, giving them more opportunities to hone relevant skills. In contrast, and often at the expense of individualized attention, group-based programs offer the benefit of social support and interaction with other parents (Mathijs et al., 2024; Mytton et al., 2014). For families with fewer concerns about child emotional and behavioral problems, the group structure might offer a unique oppourtunity to fully reap the benefits of learning from group leaders and the experiences of other families. It may also be that mothers are particularly sensitive to the peer support offered in a group setting, such that the individual telehealth format was not superior to the in-person group format regardless of initial risk. Some research has documented stronger impracts of group-based programs (versus individual ones) for mothers (e.g., Coren et al., 2003), but whether this effect is stronger for mothers than fathers is unknown due to the paucity of fathers represented in intervention research.

In terms of parents’ initial mental health symptoms, fewer significant effects emerged, but results trended in the same direction as those for child adjustment. Mothers’ initial mental health did not significantly moderate the effects of program format on parenting. Among fathers, for those lower in initial depressive symptoms, the in-person group condition was associated with greater improvements in parenting than the individual telehealth condition. In contrast, for fathers higher in initial depressive symptoms, the individual telehealth condition outperformed the in-person group condition in terms of improved parenting. The superiority of individual telehealth over in-person groups was observed at the marginal level for fathers high in initial post-traumatic stress symptoms as well. This finding converges with prior literature suggesting that within group-based ADAPT, fathers with clinical levels of PTSD showed blunted improvements in parenting compared to those without PTSD (Chesmore et al., 2018). Participation in longer, group-based sessions may have posed a challenge for fathers with more pronounced mental health challenges (e.g., social avoidance; Ottenbreit & Dobson, 2004), whereas individual telehealth likely provides a more approachable, familiar and individually-tailored environment. Taken together, the results suggest that fathers with greater mental health challenges benefit more from individual telelealth parenting interventions than in-person group-based programs.

Overall, our results suggested that fathers at higher initial risk benefited more from an individual telehealth program, whereas mothers and fathers at lower initial risk benefited more from an in-person group-based program. Findings were mirrored in models separated by civilian (mostly mothers) and military (mostly fathers) statuses. In addition to offering individualized attention, telehealth programs also remove several logistical barriers to participation that impact in-person group attendance, such as the need for transportation and childcare, inflexible work schedules, and longer distances to get to the implementation site (Dumas et al., 2007; Morawska & Sanders, 2006). It is likely, due to systemic inequities in access to resources, that families at initially higher mental health and child maladjustment risk are also those who face more barriers to in-person program access (Fernandez & Eyberg, 2009; Kazdin & Wassell, 2000). Families randomized to the telehealth condition indeed attended a greater proportion of ADAPT sessions than those in the in-person group condition (Cai et al., 2024), meaning they may have had more opportunities to learn and practice skills. Understanding the interplay between contextual factors and session engagement as it relates to program effectiveness should be a topic of future research.

### Distal Program Effects on Child Adjustment

A second aim of this study was to examine distal program effects on child internalizing and externalizing, consistent with SIL theory that changes in parenting drive intervention effects on child adjustment outcomes. Most studies of telehealth programs to date have focused on feasibility and short-term effects, with few examining long-term outcomes. For instance, in McLean and colleagues’ review of telehealth family therapy programs, only a handful of studies had follow-up timepoints beyond 6 months (McLean et al., 2021). Capitalizing on a two-year longitudinal design, we sought to understand whether program-driven improvements in parenting were related to child internalizing and externalizing. We found that improvements in mothers’ observed parenting were associated with small to moderate improvements in child internalizing and externalizing problems, consistent with prior reviews of parenting programs (Lundahl et al., 2006; Mingebach et al., 2018), whereas improvements in fathers’ parenting were not associated with change in child adjustment. Fathers, who were typically the deployed parent in this sample, may have fewer opportunities to influence children’s behavior due to less time spent together (as a result of their deployment), or children’s reliance on their non-deployed parent even after the other parent’s reintegration (Strong & Lee, 2017; Walsh et al., 2014). Indeed, in post-hoc models, changes in parenting were associated with changes in child adjustment among civilian parents, but not among previously deployed parents. The impact of specific deployment factors, such as length, recency, and child age at parents’ deployment, should be further investigated in order to inform intervention approaches for this population. Findings provide support for the positive long-term impact of the ADAPT program but also suggest that further investigation is needed to understand which parent, child, and family characteristics may lead to greater or lesser improvements in child adjustment.

#### Implications

Our findings point to the differential suitability of specific intervention formats for families characterized by different levels and types of initial risk. In particular, and relative to individual telehealth formats, in-person, group-based programs may be more fitting for parents who perceive that their children are relatively well-adjusted. In contrast, for parents – especially fathers and those who have been deployed – who perceive greater adjustment problems, as well as their own mental health challenges, individual telehealth programs may be particularly beneficial. Our findings add important nuance to decades of research among mostly in-person group-based programs that suggests that families at higher initial risk benefit more from preventive interventions (Gardner, 2023; Nowak & Heinrichs, 2008). This consensus likely holds true overall: those at greater initial risk stand to benefit more from preventive interventions generally. However, the current findings also indicate that specific formats differentially meet the needs of relatively higher or lower risk families. If replicated among other interventions and populations, these insights can serve to guide program providers in tailoring program modalities to meet families’ unique needs.

Findings also supported distal program impacts on child adjustment both directly, and indirectly, through improved parenting, regardless of program format. The small to moderate reductions documented in this study are meaningful, considering that without intervention, elevated maladjustment among children in military families would be expected (Lester et al., 2016; Trautmann et al., 2015). Whereas mothers’ (and civilian parents’) parenting was more closely associated with changes in child maladjustment problems, it was fathers’ initial risk that was more often associated with differential effects by program format. Thus, the findings suggest broader efforts to disseminate and implement the ADAPT program, in both group-based and telehealth formats, will be worthwhile in serving the needs of military parents and their children.

### Limitations and Future Directions

Despite the strengths of a longitudinal, randomized design and incorporation of multiple informants, this study is not without limitations. The key limitation is the confounding of both group vs. individual and telehealth vs. in-person program formats. Thus we cannot disentangle whether higher risk families benefited more because of the individual approach, or the telehealth modality, and vice-versa, whether lower risk families benefited more from the group-related aspects, or the face-to-face aspects. A comparative effectiveness trial examining individual vs. group in-person and individual vs. group telehealth formats would enable clear conclusions about whether the individual attention or the convenient format most benefited higher risk families. Furthermore, this comparative effectiveness study lacked a no-treatment control group, meaning the impact of specific program formats is only understood relative to other formats, rather than in terms of overall effect sizes. There may additionally be mono-informant bias in that parents self-reported their perceptions of child adjustment problems and mental health at each timepoint. However, the concerns of mono-informant bias are somewhat reduced by our use of observational parenting scores at Time 3, providing a more conservative approach than relying on parent self-reports. Future research will benefit from incorporating multiple informants and additional methods. Furthermore, understanding the impact of parents’ psychopathology on not only their own parenting responses, but on their partner’s parenting, may be an important next step toward tailoring program types to the needs of families with different initial risk profiles.

## Conclusions

Telehealth interventions have become increasingly common, and the evidence base supporting them is growing (Howland et al., 2025; McLean et al., 2021; Pan et al., 2023). This study provides preliminary evidence about which families benefit most following interventions delivered via individual telehealth versus in-person groups. The findings suggest families with fewer initial mental health and child maladjustment problems may benefit most from in-person parenting groups, whereas those with higher parent mental health and child maladjustment risk, especially as perceived by fathers, may benefit most from individual telehealth approaches. Furthermore, the intervention overall was associated with small-to-medium reductions in child maladjustment two years later, via improvements in mothers’ parenting, suggesting potential long-lasting impacts. These findings add to a growing body of support for the potential advantages in terms of feasibility and effectiveness of telehealth-delivered parenting interventions.

## Supplementary Information

Below is the link to the electronic supplementary material.

Supplementary Material 1

## Data Availability

The data and code used in the current paper are available on request from the corresponding author.

## Declarations

### Trial registration

Trial was registered at clinicaltrials.gov on 5/5/2021. ClinicalTrials.gov Record 1407S52001.
