# Supplementary material for: Who Benefits More in Group-Based and Individual Telehealth Parenting Programs: Moderators and Distal Child Outcomes among Military Families
Source: Res Child Adolesc Psychopathol. 2026 Aug 26;54(5):101. doi: 10.1007/s10802-026-01496-1 (PMC13518467; doi:10.1007/s10802-026-01496-1)
Supplement: Supplementary file 1 [file 10802_2026_1496_MOESM1_ESM.docx]

**Supplemental materials for *Who Benefits More in Group-Based and Individual Telehealth Parenting Programs: Moderators and Distal Child Outcomes among Military Families***

**Table S1.** *Results from moderated mediation models among non-deployed civilian parents*

|  | Moderator | | | | | | | |
| --- | --- | --- | --- | --- | --- | --- | --- | --- |
| *Model Fit* | **Depression** | | **PTSS** | | **Child**  **Internalizing** | | **Child**  **Externalizing** | |
| Chi Square | 14.61 (*p* = .842) | | 11.84 (*p* = .774) | | 4.05 (*p* = .774) | | 7.82 (*p* = .349) | |
| CFI | 1 | | .986 | | 1 | | .994 | |
| TLI | 1 | | .944 | | 1 | | .968 | |
| RMSEA [95% CI] | 0 [.00, .05] | | .04 [.00, .11] | | .00 [.00, .08] | | .03 [.00, .12] | |
| *Path Coefficients* | $\beta$ (S.E.) | *p* | $\beta$ (S.E.) | *p* | $\beta$ (S.E.) | *p* | $\beta$ (S.E.) | *p* |
| (a) Condition (Group vs. TH) 🡪 T3 Parenting | 0.09 (.21) | .451 | 0.09 (.13) | .490 | 0.10 (.11) | .354 | 0.10 (.12) | .396 |
| (m) Moderator on path a | -0.18 (.15) | .238 | -0.27 (.24) | .262 | -0.36 (.20) | .066 | **-0.37 (.16)** | **.019** |
| (b1) T3 Parenting 🡪 T4 Child Internalizing | **-0.34 (.15)** | **.024** | **-0.34 (.16)** | **.029** | **-0.37 (.15)** | **.015** | **-0.33 (.15)** | **.028** |
| (b2) T3 Parenting 🡪 T4 Child Externalizing | -0.21(.20) | .311 | -0.25 (.20) | .205 | -0.24 (.17) | .931 | -0.24 (.19) | .199 |
| (c’1) Condition 🡪 T4 Child Internalizing | 0.01 (.09) | .903 | 0.01 (.09) | .918 | 0.01 (.08) | .870 | 0.01 (.09) | .916 |
| (c’2) Condition 🡪 T4 Child Externalizing | 0.002 (.09) | .982 | 0.01 (.09) | .922 | 0.01 (.08) | .931 | 0.01 (.09) | .915 |
| *Autoregressive Paths* |  |  |  |  |  |  |  |  |
| T1 Parenting 🡪 T3 Parenting | **0.24 (.10)** | **.024** | **0.26 (.10)** | **.009** | 0.18 (.11) | .093 | **0.27 (.10)** | **.005** |
| T1 Child Internalizing 🡪 T4 Internalizing | **0.46 (.08)** | **<.001** | **0.47 (.08)** | **<.001** | **0.48 (.08)** | **<.001** | **0.46 (.08)** | **<.001** |
| T1 Child Externalizing 🡪 T4 Externalizing | **0.64 (.08)** | **<.001** | **0.64 (.08)** | **<.001** | **0.64 (.08)** | **<.001** | **0.64 (.08)** | **<.001** |
| *Note: :* Standardized coefficients are presented; TH = Telehealth condition; Models controlled for prior levels of all dependent  variables; Covariates: child age, child gender, intervention site, income, marital status, number of deployments;  CFI=Comparative Fit Index, TLI=Tucker-Lewis Index, RMSEA= Root Mean Square Error of Approximation | | | | | | | | |

**Table S2.** *Results from moderated mediation models among previously deployed parents*

|  | Moderator | | | | | | | |
| --- | --- | --- | --- | --- | --- | --- | --- | --- |
| *Model Fit* | **Depression** | | **PTSS** | | **Child**  **Internalizing** | | **Child**  **Externalizing** | |
| Chi Square | 9.47 (*p* = .489) | | 6.09 (*p* = .807) | | 5.65 (*p* = .931) | | 2.65 (*p* = .915) | |
| CFI | 1 | | 1 | | 1 | | 1 | |
| TLI | 1 | | 1 | | 1 | | 1 | |
| RMSEA [95% CI] | .00 [.00, .08] | | .00 [.00, .06] | | .00 [.00, 09] | | .00 [.00, .04] | |
| *Path Coefficients* | $\beta$ (S.E.) | *p* | $\beta$ (S.E.) | *p* | $\beta$ (S.E.) | *p* | $\beta$ (S.E.) | *p* |
| (a) Condition (Group vs. TH) 🡪 T3 Parenting | -0.11 (.09) | .225 | -0.11 (.10) | .252 | -0.05 (.09) | .535 | -0.07 (.09) | .431 |
| (m) Moderator on path a | **-0.45 (.16)** | **.005** | **-0.42 (.20)** | **.033** | **-0.41 (.14)** | **.004** | **-0.37 (.12)** | **.002** |
| (b1) T3 Parenting 🡪 T4 Child Internalizing | 0.05 (.11) | .621 | 0.03 (.11) | .788 | 0.02 (.11) | .867 | 0.02 (.11) | .982 |
| (b2) T3 Parenting 🡪 T4 Child Externalizing | -0.04 (.11) | .720 | -0.05 (.12) | .683 | -0.06 (.11) | .569 | -0.09 (.11) | .421 |
| (c’1) Condition 🡪 T4 Child Internalizing | -0.01 (.07) | .925 | -0.01 (.07) | .897 | -0.01 (.07) | .888 | -0.01 (.07) | .916 |
| (c’2) Condition 🡪 T4 Child Externalizing | 0.01 (.06) | .932 | 0.01 (0.06) | .934 | 0.01 (.07) | .921 | 0.01 (.07) | .926 |
| *Autoregressive Paths* |  |  |  |  |  |  |  |  |
| T1 Parenting 🡪 T3 Parenting | **0.33 (.12)** | **.004** | **0.35 (.12)** | **.004** | **0.26 (.09)** | **.006** | **0.34 (.12)** | **.003** |
| T1 Child Internalizing 🡪 T4 Internalizing | **0.62 (.06)** | **<.001** | **0.62 (.06)** | **<.001** | **0.62 (.06)** | **<.001** | **0.62 (.06)** | **<.001** |
| T1 Child Externalizing 🡪 T4 Externalizing | **0.69 (.06)** | **<.001** | **0.69 (.06)** | **<.001** | **0.69 (.06)** | **<.001** | **0.70 (.06)** | **<.001** |
| *Note:* Standardized coefficients are presented; TH = Telehealth condition; Models controlled for prior levels of all dependent  variables; Covariates: child age, child gender, intervention site, income, marital status, number of deployments;  CFI=Comparative Fit Index, TLI=Tucker-Lewis Index, RMSEA= Root Mean Square Error of Approximation; | | | | | | | | |
